# Synthesis, spectroscopic, thermal, crystal structure properties, and characterization of new Hofmann-T_d_-type complexes with 3-aminopyridine

**DOI:** 10.3906/kim-2101-32

**Published:** 2021-06-30

**Authors:** Zeki KARTAL, Onur ŞAHİN

**Affiliations:** 1 Retired Professor of Atomic and Molecular Physics, Kütahya Türkiye; 2 Department of Occupat Health & Safety, Faculty of Health Sciences, Sinop University, Sinop Türkiye

**Keywords:** Hofmann-T_d_-type complexes, 3-aminopyridine, vibration spectra, SC-XRD analysis

## Abstract

In this study, synthesis of two new tetracyanocadmate(II) and tetracyanozincate(II) complexes based on 3-aminopyridine (3AP) and investigation of their structural properties were reported. These complexes were characterized by using vibration spectroscopy, elemental, thermal analysis and single crystal X-ray diffraction (SC-XRD) techniques. Investigation of the elemental, spectral and single crystal data of these complexes showed that the formulas [Cd(3AP)_2_Zn(μ_4_-CN)_4_]_n_ (**1**) and [Cd(3AP)_2_Cd(μ_4_-CN)_4_]_n_ (**2**) fully explained their crystal structure. General information about the structural and chemical properties of these complexes obtained in single crystal form was obtained by observing the changes in the characteristic peaks of the 3AP with the [Zn(μ_4_-CN)_4_]^2-^ and [Cd(μ_4_-CN)_4_]^2-^ structures that make up these complexes. The behaviors of these complexes against changes in temperature were obtained by examining the temperature-dependent changes of their mass. The asymmetric unit of the heterometallic complexes 1 and 2 consist of half Cd(II) ion, half M ion [M = Zn1 in **1** and Cd2 in **2**], two cyanide ligands and one 3AP.

## 1. Introduction

In 1897, a young scientist named Karl Andreas Hofmann (1870–1940) invented an interesting chemical compound that would later be called his name. This interesting compound, first obtained by Hofmann, was named “Hofmann type clathrate” [1]. In order for a chemical compound to be defined as a clathrate, it must have two components: one called “host structure” and the other “guest”. The host structure is a structure consisting of various ligand molecules and transition metal atoms that make up it, and has spaces in different volumes. Guest molecules are molecules with in a generally aromatic structure that can enter the spaces of the host structure in various volumes [1–4].

The clathrates, from the day they were first discovered, continue to be of an increasing interest even today because the clathrates have a great say in solving problems such as the storage of hydrogen gas, storage of thermal energy, retention of atmospheric pollutants, bioactivity and superconductivity, which are among the biggest problems of humanity today [5–10].

Recently, new clathrates with many properties such as semiconductor clathrates, magnetic resonance imaging clathrates and electrically sensitive organic clathrates have been obtained besides clathrates with traditional properties [5–10]. 

The clathrates are named in many different ways according to their origination or the areas in which they are used. The most commonly known clathrates are clathrate hydrates, organic clathrates, inorganic clathrates, Hofmann-type clathrates, Hofmann-T_d_-type clathrates and Werner-type clathrates.

The general formula of Hofmann type clathrates synthesized in organic and inorganic studies in many scientific fields is M(II)LMʹ(II)(CN)_4_.nG. In this formula, the M and Mʹ denote +2 valence transition metal atoms and the L denotes one bidentate or two monodentate ligand molecules, G indicates a guest molecule that enters the Hofmann type host structure. Finally, “n” indicates the number of guest molecule in the host structure [2–4]. 

A clathrate can be synthesized in two ways. In one of these, all metal atoms, ligand molecules, and guest molecules that will form a clathrate are brought together in the same chemical reaction. In the other way, the host structure of the clathrate is firstly formed and then the guest molecule is placed in the spaces of the host structure. While there is only one clathrate obtained in the first of these synthesis ways, clathrates in the number and type of guest molecules that can be prisoner into the host structure obtained in the second can be obtained. Therefore, it is much more important to obtain the host structure of the clathrate first than to obtain a clathrate.

Some of the uses of Hofmann-type complexes and clathrates can be briefly listed as follows: protecting the environment from the effects of various toxic and radioactive substances, separating molecules of certain sizes from others, obtaining drinking water from seawater, making new batteries more useful than old batteries, making advanced chemical sensors, obtaining hydrogen gas economically, obtaining stronger magnetic materials to store electrical energy in the smallest possible volumes, obtaining new compounds that show superconductivity at normal temperatures to reduce losses in electrical conduction, etc.

The general formula of Hofmann type host compounds is given as M(II)LMʹ(II)(CN)_4_. If the Mʹ atom in this formula is nickel, palladium and platinum, the structure of the [Mʹ(II)(CN)_4_]^2-^ group is square planar. Such compounds are called “Hofmann type compounds” (Briefly shown as HTCs). If the Mʹ atom is zinc, cadmium and mercury, the structure of the [Mʹ(II)(CN)_4_]^2-^ group is tetrahedral. Such compounds are also called “Hofmann-T_d_-type compounds” (Briefly shown as HTDTCs). When scientific publications about Hofmann compounds are examined, it is seen that there are much more studies on HTCs, while less studies on HTDTCs.

In the past years, we obtained four new HTCs using cobalt(II), copper(II), zinc(II) and cadmium(II) atoms as transition metal atoms and 3-aminopyridine as ligand molecule [11 and 12]. In this study, we wanted to obtain two new HTDTCs in crystalline form using cadmium(II) as transition metal atom, 3-aminopyridine as ligand molecule, and [Mʹ(μ_4_-CN)_4_]^2-^ anions [Mʹ = Zn(II) and Cd(II)]. Some of the scientific studies on HTDTCs from past to present are listed in order of date [13–25].

If a chemical compound has a closed formula in the form of (C_5_H_6_N_2_) and consists of the NH_2_ group attached to the pyridine ring at the meta position, this chemical compound is called 3-aminopyridine (3AP). Other aminopyridine compounds are 2-aminopyridine (2AP) and 4-aminopyridine (4AP). All of the aminopyridines are frequently used in medicine and to obtain new chemical compounds. A great deal of information can be found in the studies of our and other researchers about aminopyridines, which are very important compounds in the field of science and technology [11,12,26–36].

Generally, cyanometallate compounds are a group of anions formed by a metal atom and cyanide ligands. There are four cyanide groups per metal atom in tetracyanometallate compounds. If the metal atoms in tetracyanomethalates are nickel, palladium, and platinum, they have a square planar geometry, and if the metal atoms are zinc, cadmium and mercury, they have a tetrahedral geometry. In a chemical structure with uniform tetrahedral geometry, there are four equally spaced sp^3^ hybrids orbital that form bond angles of approximately 109.5° between them [37].

In this study, to obtain HTDTCs with the formula Cd(3AP)_2_M(CN)_4_, the 3AP, cadmium(II) acetate monohydrate [Cd(OOCCH_3_)_2_·H_2_O] and potassium tetracyanometallate(II) K_2_[M(CN)_4_] [M = Zn(II) and Cd(II)] compounds were used. As a result of our studies, two new HTDTCs in crystal form were obtained, whose chemical formulas are considered to be Cd(II)(3AP)_2_Zn(CN)_4 _and Cd(II)(3AP)_2_Cd(CN)_4_.

## 2. Experimental

### 2.1. Syntheses of HTDTCs Cd(II)(3AP)_2_Zn(CN)_4_ and Cd(II)(3AP)_2_Cd(CN)_4_

The chemical substances used in this study were not processed any further. These chemicals are listed below.

a) 3-aminopyridine; 3AP (C_5_H_6_N_2_, (Alfa Aesar Thermo Fisher Scientific Chemicals, Inc)

b) Cadmium(II) acetate monohydrate; [Cd(OOCCH_3_)_2_·H_2_O, Alfa Aesar, 99%]

c) Potassium tetracyanozincate(II); K_2_[Zn(CN)_4_]: (It was synthesized by us.) 

d) Potassium tetracyanocadmate(II); K_2_[Cd(CN)_4_]: (It was synthesized by us.)

The following chemical analysis method was used to obtain compound Cd(II)(3AP)_2_Zn(CN)_4_. First, 1 mmol of K_2_[Zn(CN)_4_] (0.248 g) was dissolved in distilled hot water (10 mL), and 2 mmol of 3AP (0.188 g) was added to this solution. Then, a solution of 1 mmol Cd(OOCCH_3_)_2_·H_2_O (0.249 g) in distilled hot water (5 mL) was added to the mixture. The entire mixture was mixed with magnetic stirrer for about an hour. 

Similarly, the following chemical analysis method was used to obtain compound Cd(II)(3AP)_2_Cd(CN)_4_. First, 1 mmol of K_2_[Cd(CN)_4_] (0.295 g) was dissolved in distilled hot water (10 mL), and 2 mmol of 3AP (0.188 g) was added to this solution. Then, a solution of 1 mmol Cd(OOCCH_3_)_2_·H_2_O (0.249 g) in distilled hot water (5 mL) was added to the mixture. The entire mixture was mixed with magnetic stirrer for about an hour.

As a result of all these chemical reactions, HTDTCs, which are thought to be their formulas as Cd(II)(3AP)_2_Zn(CN)_4_ and Cd(II)(3AP)_2_Cd(CN)_4_ were formed in suspension form in aqueous media. The diluted ammonia solution was added to the resulting complexes to obtain cleaner and more transparent mixtures. These transparent and clear mixtures were stirred with magnetic stirrer for 3 h at approximately 55 °C and filtered to remove impurities in them and allowed to crystallize under normal conditions.

As a result of this study, the colorless, transparent complexes of the HTDTCs, which are thought to have the formula Cd(II)(3AP)_2_Zn(CN)_4_ and Cd(II)(3AP)_2_Cd(CN)_4_, were obtained after a period of about four or six weeks.

Based on the elemental analysis of the structure of these crystalline HTDTCs and the results of the SC-XRD studies, it was found that their structures are polymeric and their formulas are [Cd(3AP)_2_Zn(μ_4_-CN)_4_]_n_ ( **1** ) and [Cd(3AP)_2_Cd(μ_4_-CN)_4_]_n_ ( **2** ), respectively, as expected. 

### 2.3. Instrumentation

FT-IR spectra of the complexes **1** and **2** were obtained with the Bruker Optics Vertex 70 FT-IR Spectrometer (Bruker Optics, Ettlingen, Germany) in the wavenumber range of (3750–250) cm^–1^ at 2 cm^–1^ resolution using the KBr technique under normal laboratory conditions. FT-Raman spectrum of the complex **1** was obtained at under normal laboratory conditions with a Bruker Senterra dispersive Raman microscope using the 532-nm line of a 3B diode laser in the wavenumber range of (3750–150) cm^–1^. The FT-Raman spectrum of complex **2** could not be obtained. 

The data of the crystal structures of the complexes **1** and **2** were collected with a D8-QUEST diffractometer equipped with a graphite-monochromatic Mo-K_α_ (λ = 0.71073 Å) radiation. The H atoms of carbon atoms were located from different maps and then treated as riding atoms with C-H distance of 0.93 Å. Other H atoms were refined freely. The following procedures were implemented in our analysis: solved by direct methods; SHELXS-2013 [38]; refined by full-matrix least-squares methods; SHELXL-2013 [39]; data collection: Bruker APEX2 [40]; program used for molecular graphics were as follows: MERCURY programs [41]; software used to prepare material for publication: WinGX (Hilton Software, Coral Springs, FL, USA) [42]. Details of data collection and crystal structure determination are given in Table 1. 

**Table 1:** Crystal data and structure refinement parameters for complexes 1 and 2.

Crystal data	1	2
Empirical formula	C14H12CdN8Zn	C7H6CdN4
Formula weight	470.09	258.56
Crystal system	Monoclinic	Monoclinic
Space group	C2/c	C2/c
a (Å)	13.698 (3)	14.203 (3)
b (Å)	9.882 (2)	9.736 (2)
c (Å)	13.360 (3)	13.844 (3)
b (º)	92.330 (6)	91.969 (7)
V (Å3)	1807.1 (7)	1913.3 (7)
Z	4	8
Dc (g cm–3)	1.728	1.795
μ (mm–1)	2.52	2.23
θ range (º)	2.9 - 28.3	2.9 - 28.4
Measured refls.	25591	22895
Independent refls.	1767	1869
Rint	0.037	0.032
S	1.13	0.91
R1/wR2	0.026 / 0.077	0.023 / 0.051
Drmax/Drmin (eÅ–3)	1.25 / -0.47	1.12 / –0.45

The thermal curves of complexes **1** and **2** were recorded in a nitrogen environment at a heating rate of 5 ^o^C/min and in the temperature range (25–600) ^o^C using platinum crucibles on a SETARAM LabSys evo (SETARAM Instrumentation, Caluire, France) thermal analyzer. 

For this study, the metal amounts in the structure of the complexes obtained were analyzed with the Perkin-Elmer optima 4300 DV ICP-OES device (PerkinElmer, Inc., Waltham, MA USA), and the carbon, nitrogen and hydrogen amounts were analyzed with the CHNS-932 (LECO Corporation, St. Joseph, MI, USA) elemental measuring device. The results obtained from these measurements are shown in Table 2.

**Table 2:** Elemental analysis of complexes 1 and 2.

The HTDTCs and molecular weight Mr (g)	Elemental analysis, Found(%)/(Calculated)(%)				
	C	H	N	Zn	Cd
[Cd(3AP)2Zn(μ4-CN)4]n; Mr = 470.10	36.93(35.77)	2.45(2.57)	23.19(23.84)	13.63(13.91)	23.49(23.91)
Cd(II)0.5(3AP)Cd0.5(CN)2; Mr = 258.56[Cd(3AP)2Cd(μ4-CN)4]n; Mr = 517.12	31.69(32.52)	2.48(2.34)	21.82(21.67)	-(-)	42.39(43.48)

From the examination of Table 2, it is seen that the theoretical computation results for complexes **1** and **2** and the experimental results are in good agreement with each other.

## 3. Results and discussion

### 3.1. Crystallographic analyses of complexes 1 and 2

**Figure 1:**
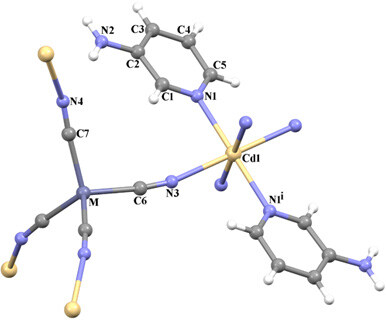
The molecular structures of complexes 1 and 2 [M = Zn1 in 1 and Cd2 in 2] showing the atom numbering schemes [(i) 1/2-x, 3/2-y, 1-z for 1 and [(i) 1/2-x, 1/2-y, 1-z for 2].

**Figure 2:**
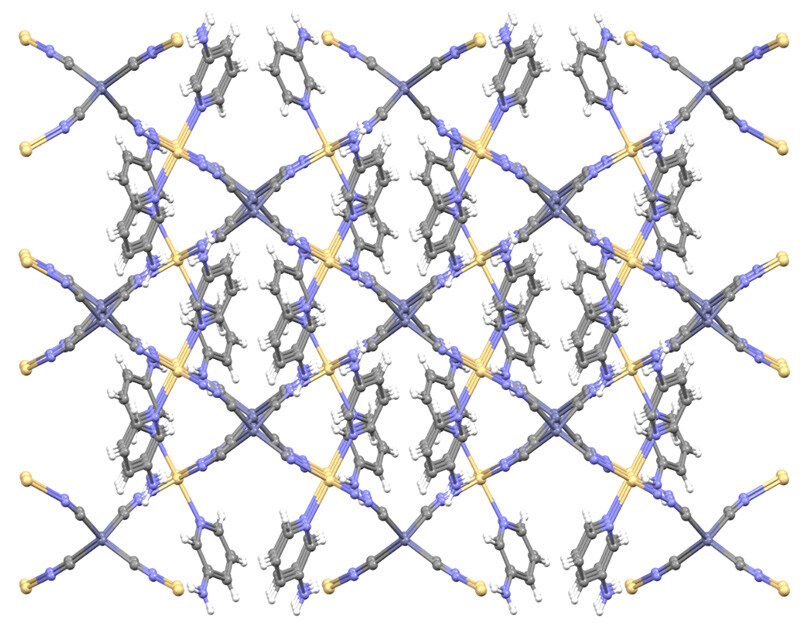
An infinite 3D structure in 1 and 2.

The SC-XRD study shows that heterometallic complexes **1** and **2** have 3D coordination polymers. Each complex crystallizes in the monoclinic system with space group *C2* / *c* . While the atoms located in the symmetry centers in complex **1** are Cd and Zn atoms, the metal atom located in both symmetry centers in complex **2** is Cd atoms. Therefore, the formula for complex **2** gives half the formula for complex **1** (see Table 1). The asymmetric unit of the heterometallic complexes **1** and **2** consist of half Cd(II) ion, half M ion [M = Zn1 in **1** and Cd2 in **2** ], two cyanide ligands and one 3AP as shown in Figure 1. In complexes **1** and **2** , each Cd1 atom is located on a center of symmetry and is coordinated by four nitrogen atoms from cyanide ligands and two nitrogen atoms from 3AP [Cd1-N bond range between 2.323(3) and 2.327(3) Å in **1** and 2.339(3) and 2.313(3) Å in **2** ], thus showing a distorted octahedral coordination geometry. The two 3AP and four CN groups are located in an octahedral structure with respect to the Cd1 atom and in a trans position. These Cd-N distances were found by different researchers to be 2.323(7), 2.319(3) and 2.335(2) Å, respectively [43–45]. The bond distance between the Cd1 atom and the nitrogen atom of the cyanide ligand [2.399(3) Å in **1** and 2.381(3) Å in **2]** was found by different researcher to be 2.207(8) Å [43]. Each M ion [M = Zn1 in **1** and Cd2 in **2** ] is coordinated by four carbon atoms from cyanide ligands [2.037(3) and 2.038(3) Å in **1** and 2.211(3) and 2.218(3) Å in **2** ], thus showing a tetrahedral coordination geometry. These M-C distances were found to be 2.020(4) and 2.207(8) Å in crystal structures by other researchers with same metal atoms and cyanide ligands [43,46]. The metal ions are bridged by cyanide ligands to generate 3D coordination polymers as shown in Figure 2, with the Cd1∙∙∙Zn1 separations are 5.354 and 5.428 Å in **1** and the Cd1∙∙∙Cd2 separations are 5.485 and 5.576 Å in **2** .

**Figure 3:**
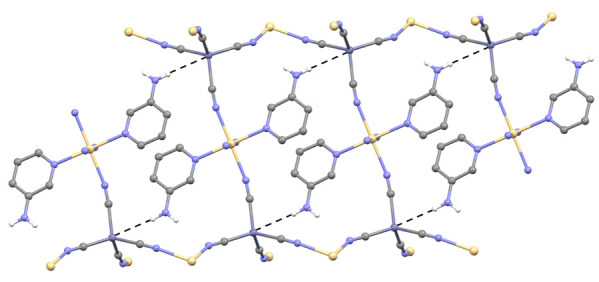
The N-H⋯M interactions [M = Zn1 in 1 and Cd2 in 2] in 1 and 2.

The weak intermolecular N–H⋯M interaction between M(II) ion and amino H atom of 3AP [H2A⋯Zn1^i^ = 3.06(2) Å, N2⋯Zn1^i ^= 3.759(1) Å and N2–H2A⋯Zn1^i ^= 139.65(3)° in **1** and H2A⋯Cd2^ii^ = 3.18(2) Å, N2⋯Cd2^ii ^= 3.780(2) Å and N2–H2A⋯Cd2^ii ^= 127.13(2)° in **2** ] is the most outstanding property of **1** and **2** [(i) x-1/2, y-1/2, z; (ii) 1-x, 1-y, 1-z]. The weak N–H⋯M interactions that occur in the crystal structures we have examined are clearly seen in Figure 3. These all type connections play a big role in making the crystal structures even stronger. In addition, such weak connections cause changes in the values of the N-H stretching and bending vibrations. The bond distances, bond angles between some selected atoms in the complexes **1** and **2** are given in Table 3.

**Table 3:** Selected bond distances and angles for complexes 1 and 2 (Å, º).

Complex 1			
Cd1-N1	2.323(3)	Cd1-N3	2.327(3)
Cd1-N4ii	2.399(3)	Zn1-C7	2.037(3)
Zn1-C6	2.038(3)	C6-N3	1.127 (4)
C7-N4	1.129 (4)		
N1-Cd1-N3i	91.32(10)	N1-Cd1-N3	88.68(10)
N1-Cd1-N4ii	88.82(11)	N3-Cd1-N4ii	88.74(11)
N1-Cd1-N4iii	91.18(11)	C7-Zn1-C7iv	114.76(18)
C7-Zn1-C6iv	111.37(12)	C7-Zn1-C6	104.22(12)
C6-N3-Cd1	167.8 (3)		
Complex 2			
Cd1-N3	2.313(3)	Cd1-N1	2.339(3)
Cd1-N4ii	2.381(3)	Cd2-C6	2.211(3)
Cd2-C7C7-N4	2.218(3) 1.129 (4)	C6-N3	1.128 (4)
N3-Cd1-N1	90.51(11)	N3-Cd1-N1i	89.49(11)
N3-Cd1-N4ii	89.33(12)	N3-Cd1-N4iii	90.67(12)
N1-Cd1-N4ii	89.98(11)	C6-Cd2-C7iv	110.28(12)
C6-Cd2-C7	102.91(12)	C7-Cd2-C7iv	116.15(17)
C6-N3-Cd1	167.7 (3)		

As can be seen from the literature review, there are only one kind of α-type cavities for guest molecules in the host structures of HTCs, while there are two kinds of α and β-type cavities for guest molecules in the host structures of HTDTCs. The α-type cavities are approximately rectangular prism-shaped structures like those in HTCs. The β-type cavities are a twisted structure formed by rotating a 90° of half of a rectangular prism cut along with diagonal plane. The metal atoms that make up the HTDTCs are located at the corners of this rectangular prism [14–25,43,46].

**Figure 4:**
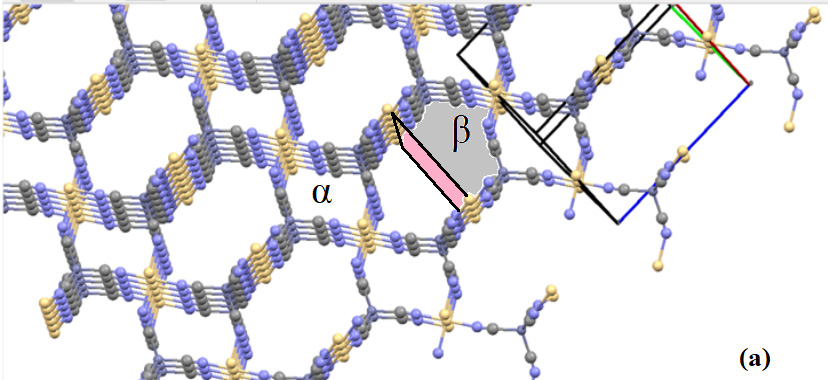

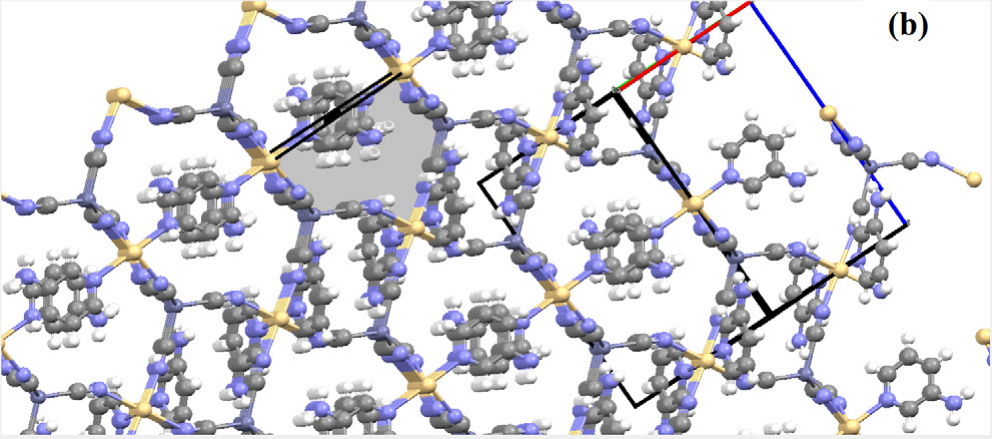
The views of α and β-type cavities occurring in complexes 1 and 2 (a) without 3AP, and (b) with 3AP.

In order to see α and β-type cavities formed in the complexes more easily, the lattice structure of a complex was created without considering the 3AP ligand molecule in the crystal structure analysis program. It has been observed that α and β- type cavities in this newly formed lattice structure are determined more easily. The resulting situation can be seen in Figure 4. Thus, it was seen how 3AP settled in α and β-type cavities. If these complexes are intended to be used as a clathrate, appropriately sized guest molecules can enter the remaining volumes of α and β-type cavities. 

### 3.2. Spectral characterization of complexes 1 and 2

**Figure 5:**
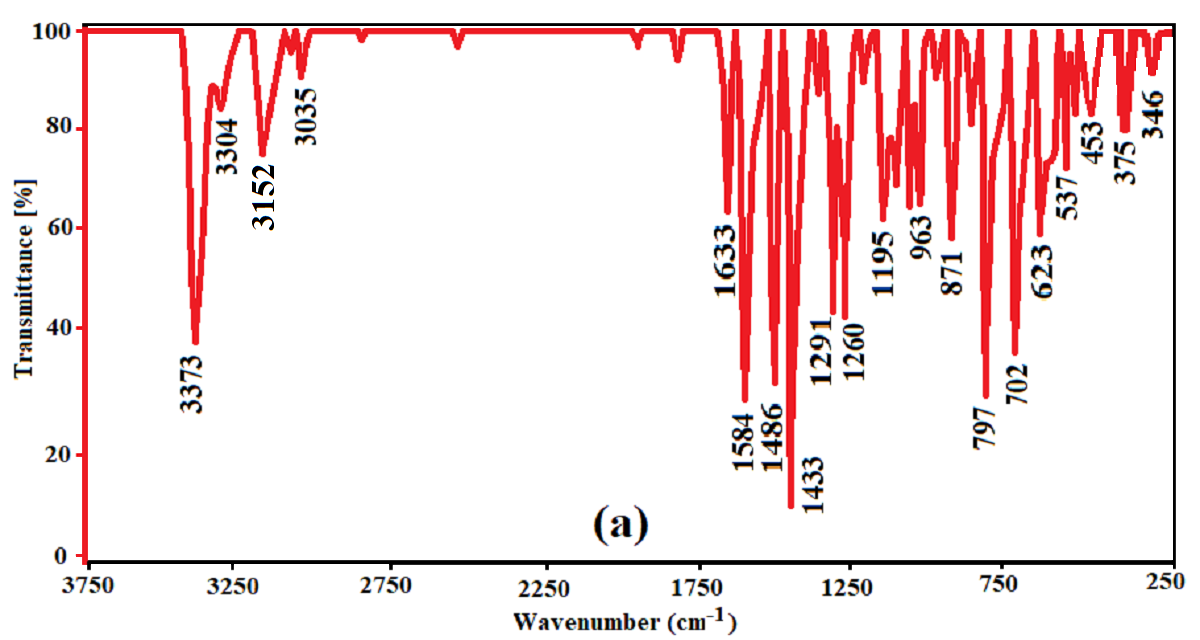

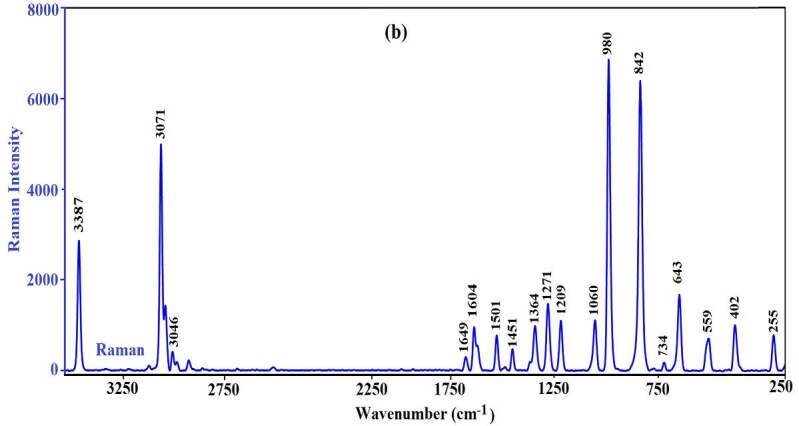

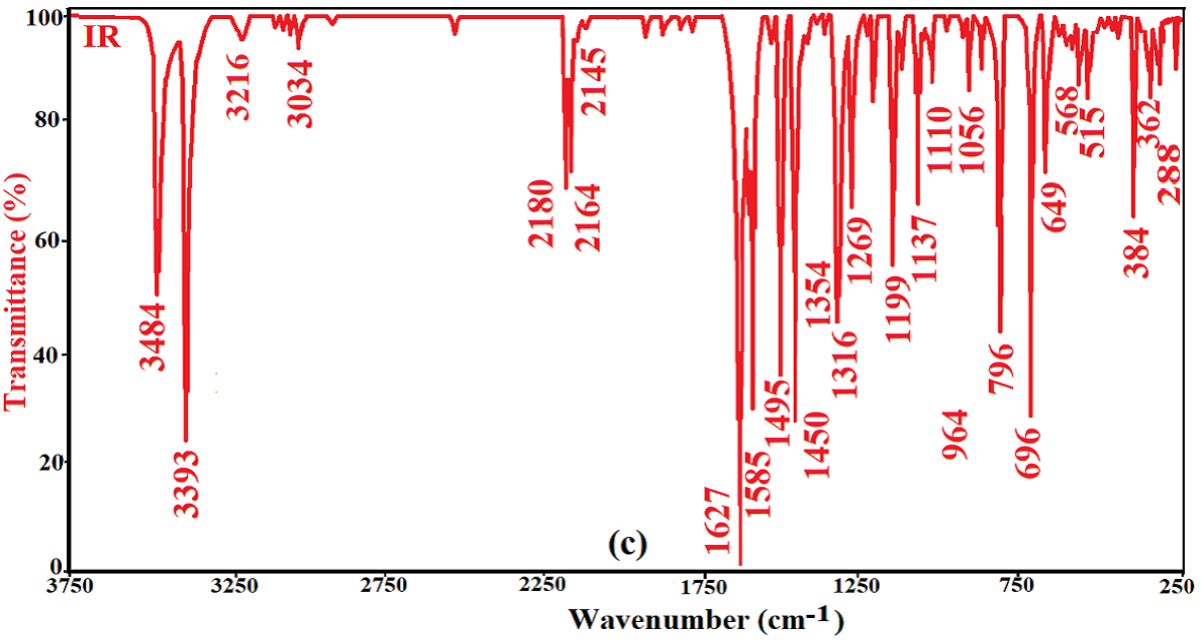

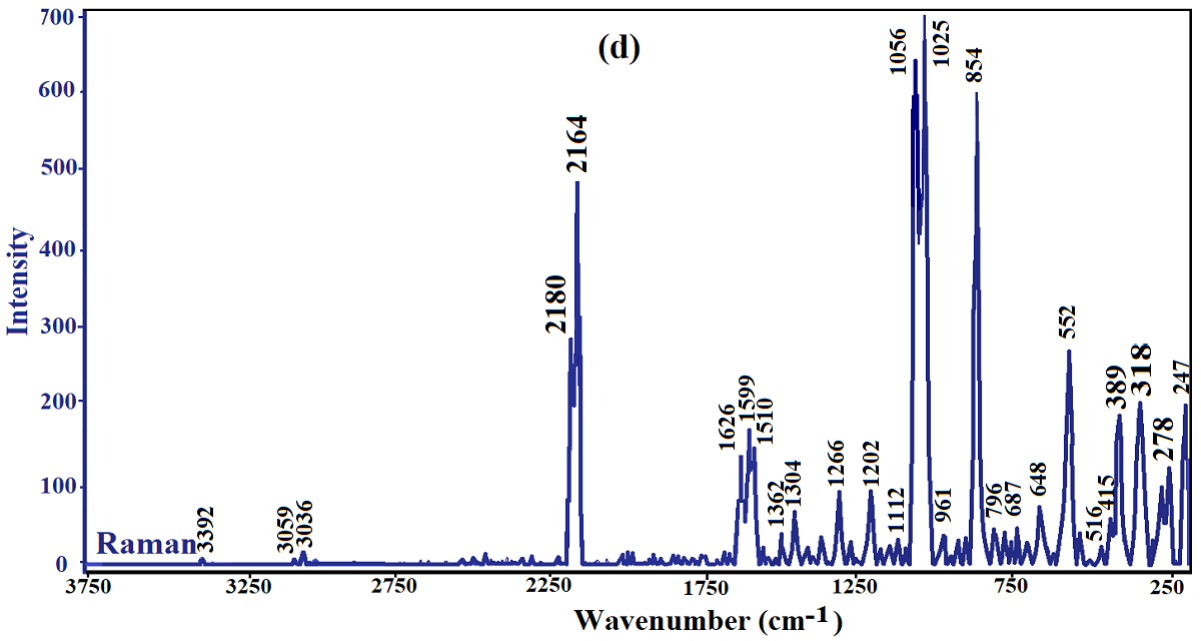

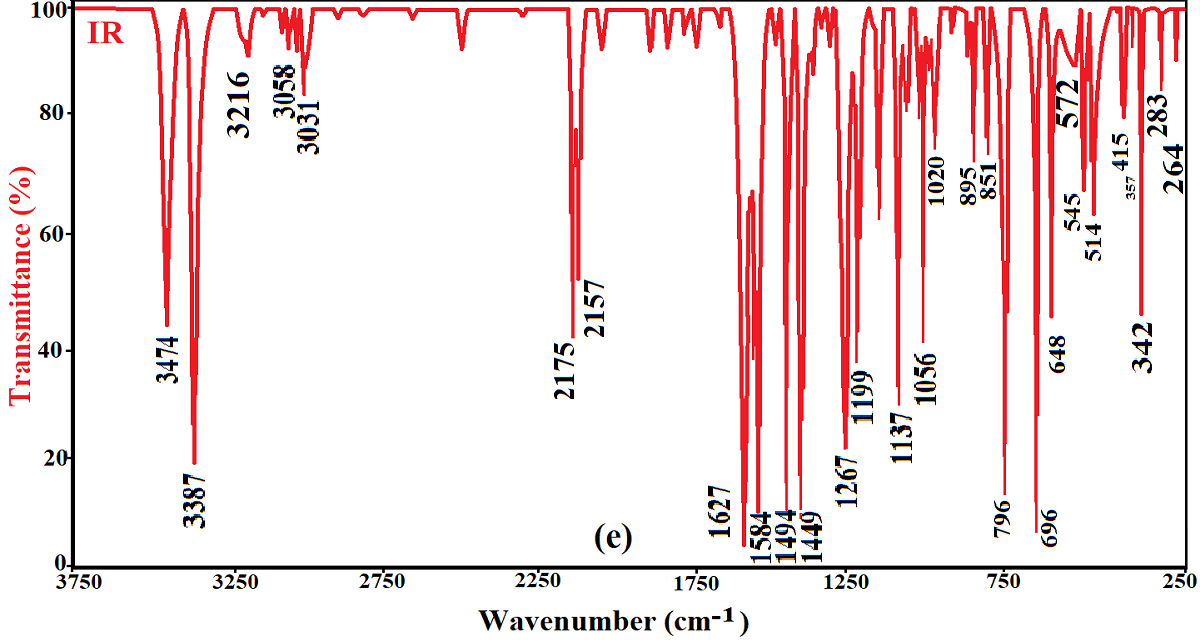
The vibrational spectra of 3AP (a), (b); complex 1 (c), (d) and FT-IR spectra of complex 2 (e).

The vibration (FT-IR and FT-Raman) spectra of 3AP are shown in Figures 5 (a), 5 (b); complex **1** 5 (c), 5 (d) and FT-IR spectra of complex **2** 5 (e), respectively. The FT-Raman spectrum of complex **2** could not be obtained due to technical reasons.

From examining the spectra of complexes **1** and **2** , the great similarities between them are immediately apparent. This shows that the structures of both complexes are similar. The presence of vibration bands of the 3AP in the vibration spectra of complex **1** and **2** is the greatest evidence of the presence of the 3AP in their structures. Spectral data of the complexes can be analyzed separately for vibrations of the 3AP, [Zn(μ_4_-CN)_4_]^2-^ and [Cd(μ_4_-CN)_4_]^2-^ anions, respectively.

#### 3.2.1. Vibrations of the 3AP 

There are many scientific studies conducted by various researchers in the past about the 3AP [11,12 and 26-36]. The 3AP has a planar structure and it belongs to the *C* *_s_* [28–30] symmetry. The 3AP has 33 normal vibration modes [29,47]. The 3AP has unshared electron pairs on the nitrogen atoms of the pyridine ring and NH_2_ group. Thus, the 3AP can be function as a bidentate ligand molecule [28–30,47].

However, when the structures of complexes **1** and **2** are examined, it is seen that the 3AP is bound only from the nitrogen atom in the pyridine ring to the cadmium transition metal atom [12,34]. Similar binding pattern has also been seen in studies with the 4AP ligand molecule [48,49]. 

Some noticeable small frequency shifts of the 3AP in the vibration spectra of complexes **1** and **2** are thought to be due to changes in environmental conditions compared to the case where the 3AP does not form compounds due to the formation of compounds. In addition, small changes occur in some vibrations due to the interaction of vibrations of the 3AP and the vibrations of the metal nitrogen bond. However, significant changes are observed in the stretching, scissoring, bending, and torsion frequencies of the NH_2_ group and ring breathing modes of the 3AP. The vibration frequencies of the 3AP most affected by changes in environmental conditions or the formation of complexes, and their shift amounts are marked in bold in Table 4. The letter of Δ is used to denote frequency shifts in this table. The +Δ values in this table indicate that the shift is towards the high frequency region, the -Δ values indicate that the shift is towards the low frequency region. Similar interactions have been observed in the studies previously conducted by us and other researchers with aminopyridine ligand molecules [11,12,27,31,33].

**Table 4:** The vibrational absorption wavenumbers (cm-1) of the solid 3AP in solid state and complexes 1 and 2.

No	Assignment a	3AP		1				2	
		IR	Raman	IR	Δ	Raman	Δ	IR	Δ
1	νas(NH2)	3373 s	3387w	3484m	+111	3392w	+5	3474m	+101
2	νs(NH2)	3304m	3319w	3393 s	+89	n.o.	N. d.	3387 s	+83
3	ν(C-H)	3066w	3071m	3059w		3059w		3058w	
4	ν(C-H)	3035w	3046w	3034w		3036w		3031w	
5	δ(NH2)	1633m	1649w	1627 s	–6	1626w	-23	1627 s	–6
6	νring + δ(CH)	1584 s	1604m	1585 s		1599m		1584 s	
7	νring	1486 s	1501w	1495 s	+9	1510w	+9	1494 s	+8
8	δ(CH) + νring	1433 s	1451w	1450 s	+17	1450w	-1	1449 s	+16
9	δ(CH)	1347w	1364w	1354w		1362w		1354w	
10	ν(C-NH2) + νring	1291 s	1306w	1316 s	+24	1304w	-2	1304 s	+13
11	νring	1260 s	1271w	1269m	+9	1266w	-5	1267m	+7
12	δ(CH) + νring	1195w	1209w	1199w		1202w		1199m	
13	δ(CH) + νring + δ(CNH)	1125m	1141w	1137m	+12	1135w	-6	1137 s	+12
14	NH2 twist + νring	1091m	n.o.	1110w		1112w		1111w	
15	Ring bre., νring + δ(CH) δ(CNH)	1042m	1060 s	1056w	+14	1056 s	-4	1056 s	+14
16	δring + νring	1014w	1028m	1020m	+6	1025 s	-3	1020m	+6
17	γ(CH)	963 m	980 s	964 w		961 w		966 w	
18	γ(CH)	908 w	n.o.	910 w		915 w		912 w	
19	γ(CH)	871 m	n.o.	893 w	+22	888 w	N. d.	895 m	+25
20	γ(CH) + δring + γ(C-NH2)	843 m	842 m	853 w	+10	854 w	+12	851 m	+8
21	γ(CH) + γring + γ(C-NH2)	797 s	815 m	796 s		796 w		796 s	
22	γring + γ(CH)	702 s	734 vw	696 s		687 w		696 s	
23	δring	623 m	643 w	649 m		648 m		648 m	
24	δring + NH2 wag. + γ(C-NH2)	537 w	559 w	545 w		552 m		545 w	
25	NH2 wag. + γ(C-NH2) + γring	510 w	n.o.	n.o.		n.o.		n.o.	
26	γ(C-NH2) + NH2 wag. + γring	453 w	402 w	515 w	+62	516 w	+114	514 m	+61
27	γ(C-C-C)	375 w	385 w	417 w		415 m		415 m	
28	γ(C-C-N)	346 w	375 w	362 w		389		377 vw	
29	γ(C-NH2)	-	255 w	-		247 w		-	

As can be seen from the examination of Table 4, the asymmetric stretching vibration of the NH_2_ group in the FT-IR and FT-Raman spectra of the 3AP in free state was observed as a peak at 3373 and 3387 cm^–1^ wavenumbers, respectively. This peak was observed in the FT-IR and FT-Raman spectra of complex **1** at 3484 and 3392 cm^–1^ wavenumbers, respectively. This vibration peak occurred in the FT-IR spectrum of complex **2** at 3474 cm^–1^ wavenumber. Due to the change of environmental conditions in the obtained complexes of the 3AP, this vibration mode shifted to the higher wavenumber region of 111and 101 cm^–1^ in the FT-IR spectra of complexes **1** and **2** , respectively. The same vibration mode, for the same reason, shifted to the higher wavenumber region of 5 cm^–1^ in the FT-Raman spectrum of complex **1** .

The symmetric stretching vibration of the NH_2_ group in the FT-IR and FT-Raman spectra of the 3AP in solid state was observed as a peak at 3304 and 3319 cm^–1^ wavenumbers, respectively. This peak was observed in the FT-IR spectra of complex **1** and **2** at 3393 and 3387 cm^-1^ wavenumbers, respectively. This peak wasn’t observed in the FT-Raman spectra of complex **1** . Due to the change of environmental conditions in the obtained complexes of the 3AP, this vibration mode shifted to the higher wavenumber region of 89 and 83 cm^–1^ in the FT-IR spectra of complexes **1** and **2** , respectively.

A peak was not seen in the theoretical vibration calculations of the 3AP molecule, but that peak was seen at 3152 cm^–1^ wavenumber in its free state IR spectrum, it was seen at 3216 cm^–1^ wavenumber in both complexes formed by 3AP. While this peak was interpreted by some researchers as the overtone of the bending vibration of the NH_2_ group, some other researchers interpreted this peak as the splitting of the stretching vibration of the NH_2_ group due to the Fermi resonance. Similar situations arose also for the 4AP molecule and the complexes obtained with it [48].

The bending vibration of the NH_2_ group in the FT-IR and FT-Raman spectra of the 3AP in solid state was observed as a peak at 1633 and 1649 cm^–1^ wavenumbers, respectively. This peak was observed in the FT-IR spectra of complexes **1** and **2** at 1627 cm^–1^ wavenumber. This peak was observed in the FT-Raman spectra of complex **1** at 1626 cm^–1^ wavenumbers. Due to the change of environmental conditions in the obtained complexes of the 3AP, this vibration mode shifted to the lower wavenumber region of 6 cm^–1^ in the FT-IR spectra of both complexes and 23 cm^–1^ in the FT-Raman spectrum of the complex **1** .

The changes in vibration modes in rows 7, 8, 10, 11, 13, 15, 19, 20, and 26 of Table 4 are the changes due to the formation of the compounds and only affect the vibration modes of the pyridine ring of 3AP. Among the changes in these vibration modes, those in the 19th and 26th rows are the most important. The values ​​of shifts in these modes are + 22, + 25 cm^–1^ and + 62, + 114, + 61 cm^–1^, respectively.

#### 3.2.2. Vibrations of group [M(μ4-CN)4]2- [M = Zn(II) and Cd(II)] in the complexes 1 and 2. 

The band assignments of [M(μ_4_-CN)_4_]^2-^ ions [M = Zn(II) and Cd(II)] in the vibration spectra of complexes **1** and **2** are based on Jones’ work [50]. Jones explained the structure of the [M(μ_4_-CN)_4_]^2–^ ions in K_2_[M(CN)_4_] compounds [M = Zn(II), Cd(II) and Hg(II)], considering them as isolated units in T_d_-symmetry [50]. The vibration modes of the [M(μ_4_-CN)_4_]^2–^ ion groups in the structures of complexes **1** and **2** are given in Table 5 together with the vibration modes of the [M(μ_4_-CN)_4_]^2-^ ion groups in the K_2_[M(CN)_4_] compounds [M = Zn(II) and Cd(II)].

**Table 5:** The some vibrational wavenumbers (cm-1) of [M(μ4-CN)4]2- groups [M = Zn(II) and Cd(II)] in complexes 1 and 2.

Assignment a	K2[Zn(CN)4]	K2[Cd(CN)4]	1	Δ	2	Δ
ν1(C≡N), A1	(2157)	(2149)	(2181 m) (2163 s)	(+24)(+ 6)	(-)(-)	(-)(-)
ν5(C≡N), F2	2152	2145	2180 m2164 m	+28+12	2175 s2157 s	+30+12
Hot band	N. d.	N. d.	2145 sh	N. d.	2129 sh	N. d.
ν2(M-C), A1	(347)	(327)	(389 w)	(+42)	(-)	(-)
ν6[ν(M-C)+ δ(NCM)], F2	359	316	384 s	+25	342 s	-17
ν7[ν(M-C)+ δ(NCM)], F2	315	250	347 w	+32	283 w	+33
ν9[δ(MCN)], F1	230	194	269 w	+39	-	-

When a compound is formed as a result of a chemical reaction, if new CN-M bonds are formed in that compound, as a result of the interaction of the vibrations of the CN group and the vibrations of the newly formed M-N bond, the CN stretching vibration modes shift to the high frequency region [15–24]. In this study, a result similar to the situation described above emerged (see Figures 2 and 4).

In order for a vibration mode of any chemical group to be split into more than one value, either the same group must have more than one property (such as different environmental conditions, different bonding forms etc.) or have different bond constants. The existence of different bond lengths for the same chemical group requires different bond constants for that chemical group [51].

 The splitting in these vibration modes are thought to be due to the fact that the bond lengths of the CN groups are not exactly equal to each other (hence the bond constants are different), and a small amount of distortion at the T_d_ symmetry of the [M(μ_4_-CN)_4_]^2-^ion groups. From the examination of Table 3, it can be seen that the four CN groups in the structure of [M(μ_4_-CN)_4_]^2-^ involved in the formation of complexes **1** and **2** are divided into two groups with two different bond lengths. Therefore, the bond constants of these CN groups also have two different values. Similar cases to these splitting in the vibration modes of the CN groups were encountered in the studies of other researchers [20,52]. The following conclusions can be reached from examining Table 5.

The ν_1_(C≡N), A_1_ vibration mode of the K_2_[Zn(CN)_4_] compound was observed in its Raman spectrum at 2157 cm^–1^ wavenumber. This vibration mode has appeared in the Raman spectrum of complex **1** as split into two in the wavenumbers 2181 and 2163 cm^–1^ and shifted to the high wavenumber region. The high wavenumber shift values occurring in this vibration mode were about 24 and 6 cm^–1^, respectively.

The vibration mode ν_5_(C≡N), F_2_ of K_2_[Zn(CN)_4_] and K_2_[Cd(CN)_4_] compounds were observed in their IR spectra at 2152 and 2145 cm^–1^ wavenumbers, respectively. This vibration mode appeared to split in two at 2180 and 2164 cm^–1^ wavenumbers in the IR spectrum of complex **1** , and, at 2175 and 2157 cm^–1^ wavenumbers in the IR spectrum of complex **2** , and shifted to the high wavenumber region, respectively. The high wavenumber shift values occurring in this vibration mode were about (28 and 12) cm^–1^ for complex **1** and (30 and 12) cm^-1^ for complex **2** , respectively. When Table 5 is examined, it is seen that the peaks of the other vibration modes are not split, but almost all of them shift to a certain amount of high wavenumber region.

The M-N stretching vibration peaks in the complexes synthesized by us were obtained in the IR spectra of these complexes as (568, 362 and 288) cm^-1^ for complex **1** and (572, 357 and 281) cm^-1^ for complex **2** , respectively. Similar situations to these results obtained here have been observed in previous studies by other researchers [35 and 52].

From the examination of Table 5, it appears that the newly obtained complexes **1** and **2** are new host structures similar to HTDTCs.

#### 3.2.3. The thermal behavior of complexes 1 and 2

**Figure 6:**
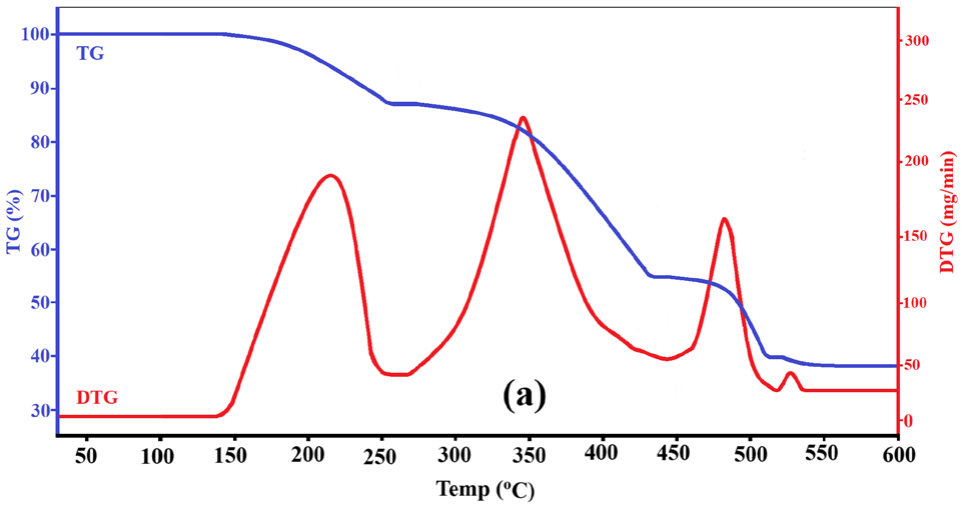

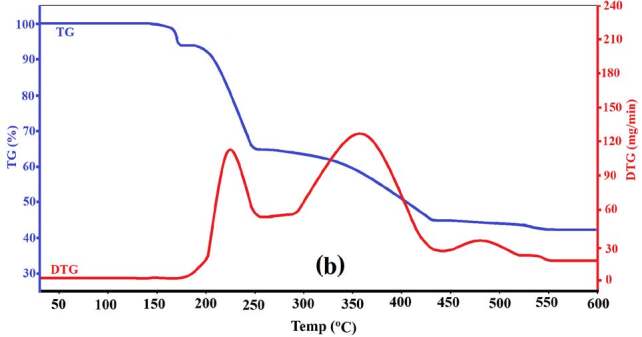
The thermal curves of complexes 1 (a) and 2 (b).

The thermal behavior of complexes **1** and **2** in response to the change in the temperature of the environment they are in was investigated in the temperature range of 25 to 600 ℃ and in a nitrogen gas environment. In this study, the thermal behavior graphs of both complexes are given in Figures 6 (a) and 6 (b), respectively. Analyzing the thermal behavior curves of complexes **1** and **2** , it was seen that no change occurred in their structure from room temperature to a certain temperature. The temperatures at which complexes **1** and **2** begin to react to the temperature increase of the environment are 142 and 162 °C, respectively. At temperature increases after the specified temperature values, the thermal behaviors of complexes **1** and **2** show a four-stage formation.

The temperature ranges of each decomposition step according to the temperature increase of the complexes, the maximum temperature value (DTG_max_) of this decomposition step, the theoretically calculated values of the product separated from the crystal structure in this decomposition step and the experimentally obtained values are given in Table 6.

**Table 6:** Decomposition steps of complexes 1 and 2 due to temperature increase.

Complexes	Thermal range, DTGmax and decomposition product in steps	Thermal decomposition steps of complexes 1 and 2			
		1st step	2nd step	3th step	4th step
1	Thermal ranges (°C)	142–249	262–425	445–506	524–540
	DTGmax (°C)	220	345	487	536
	Decomposition productFound % / (Calc.%)	NH2 group6.53 / (6.82)	Pyridine ring33.67 / (33.22)	CN group21.93 / (22.14)	Zn + Cd35.17 / (37.82)
2	Thermal ranges (°C)	162–259	266–443	447–515	525–560
	DTGmax (°C)	237	361	490	543
	Decomposition productFound % / (Calc.%)	NH2 group5.71 / (6.20)	Pyridine ring31.09 / (30.20)	CN group20.43 / (20.12)	Cd244.95 / (43.48)

As can be seen from both Figure 6 and Table 6, the 1st decomposition step occurred in the temperature ranges of 142–249 °C and 162–259 °C in complexes **1** and **2** , respectively, and NH_2_ groups were separated from the crystal structures in this decomposition step. The DTG_max_ values and the percentage amounts of NH_2_ groups separated from the crystal structures in this decomposition step are 220 to 237 °C and 6.53 / (6.82) to 5.71 / (6.20) for complexes **1** and **2** , respectively.

The 2nd decomposition step occurred in the temperature ranges of 262–425 °C and 266–443 °C in complexes **1** and **2** , respectively, and pyridine groups were separated from the structures of complexes in this decomposition step. The DTG_max_ values and the percentage amounts of pyridine groups separated from the structures of complexes in this decomposition step are 345 to 361 °C and 33.67 / (33.22) to 31.09 / (30.20) for complexes **1** and **2** , respectively.

The 3^th^ decomposition step occurred in the temperature ranges of 445–506 °C and 447–515 °C in complexes **1** and **2** , respectively, and C≡N groups were separated from the structures of complexes in this decomposition step. The DTG_max_ values and the percentage amounts of C≡N groups separated from the structures of complexes in this decomposition step are 487 to 490 °C and 21.93 / (22.14) to 20.43 / (20.12) for complexes **1** and **2** , respectively.

The last decomposition step or 4^th^ step occurred in the structures of complexes in the temperature ranges 524–540 °C and 525–560 °C, respectively. In this step, the Zn and Cd transition metal atoms forming the complexes remained in the experimental environment. DTG_max_ values ​​and the percent amounts of transition metals remaining in the experimental medium in this heat treatment step are 536 to 543 °C and 35.17 / (37.82) to 44.95 / (43.48) for complexes **1** and **2** , respectively.

## 4. Conclusion 

Two new Hofmann-(3AP)_2_-T_d_-type host structures were chemically synthesized for the first time in crystalline form. In addition, these host structures were characterized by elemental analysis, SC-XRD, vibration spectroscopy and thermal analysis techniques. It was observed that these two host structures had similar crystal properties. All vibration data of 3AP in compounds show that it is a ligand bonded from the nitrogen atom of the pyridine ring.

It is seen that the vibration spectral data and crystallographic data of these complexes support each other in a great way. Both complexes crystallized in the monoclinic crystal system and in the *C2/c* space group.

In the formation of the crystal structures of both compounds, the 3APs are bound to the Cd1 atom in trans position from the nitrogen atom of their pyridine ring. In addition, 3APs contributed to the formation of the crystal structure by bonding from a hydrogen atom of their NH_2_ group to the M atoms of the [M(μ_4_-CN)_4_]^2-^ group [M = Zn(II) in **1** and Cd(II) in **2** ], with weak hydrogen bonds.

The fact that the spectral data of the two newly acquired host complexes are similar to the spectral properties of the Hofmann-T_d_-type host structures previously obtained indicates that these new complexes are other new examples similar to the HTDTCs.

From now on, new researchers who will research on this subject can use the same ligand molecule, other transition metal atoms, and [Hg(μ_4_-CN)_4_]^2–^ ion group to create new examples of HTDTCs. They can also use other aminopyridine molecules (2AP and 4AP) or other ligand molecules to provide newer examples of HTDTCs.

## Supplementary material

Crystallographic data for the structures reported in this paper have been deposited in the Cambridge Crystallographic Data Center with CCDC numbers 2046784 for **1** and 2046785 for **2** . Copies of this information may be obtained free of charge from the Director, CCDC, 12 Union Road, Cambridge CB2 1EZ, UK (fax: +44-1223-336033; e-mail: deposit@ccdc.cam.ac.uk or www: http://www.ccdc.cam.ac.uk).
